# Converging evidence for functional connections between the lithium response and PI3K-Akt signaling

**DOI:** 10.1038/s41398-024-03160-y

**Published:** 2024-11-01

**Authors:** Donard S. Dwyer

**Affiliations:** Departments of Psychiatry and Behavioral Medicine, and Pharmacology, Toxicology and Neuroscience, LSU Health Shreveport, Shreveport, LA USA

**Keywords:** Pharmacogenomics, Bipolar disorder

Recently, Ou et al. [1] reported in *Translational Psychiatry* the replication and extension of previous findings by this group concerning mechanisms involved in the response to lithium. Collectively, GWAS, multi-omics, and network-assisted genomic analysis identified the PI3K-Akt signaling pathway as a major contributor to lithium responsiveness in bipolar disorder (BD). Importantly, these new findings confirm earlier studies from 2011 of lithium’s effects in *Caenorhabditis elegans* [2] and validate the relevance of this model organism for psychiatric research.

**Fig. 1:**
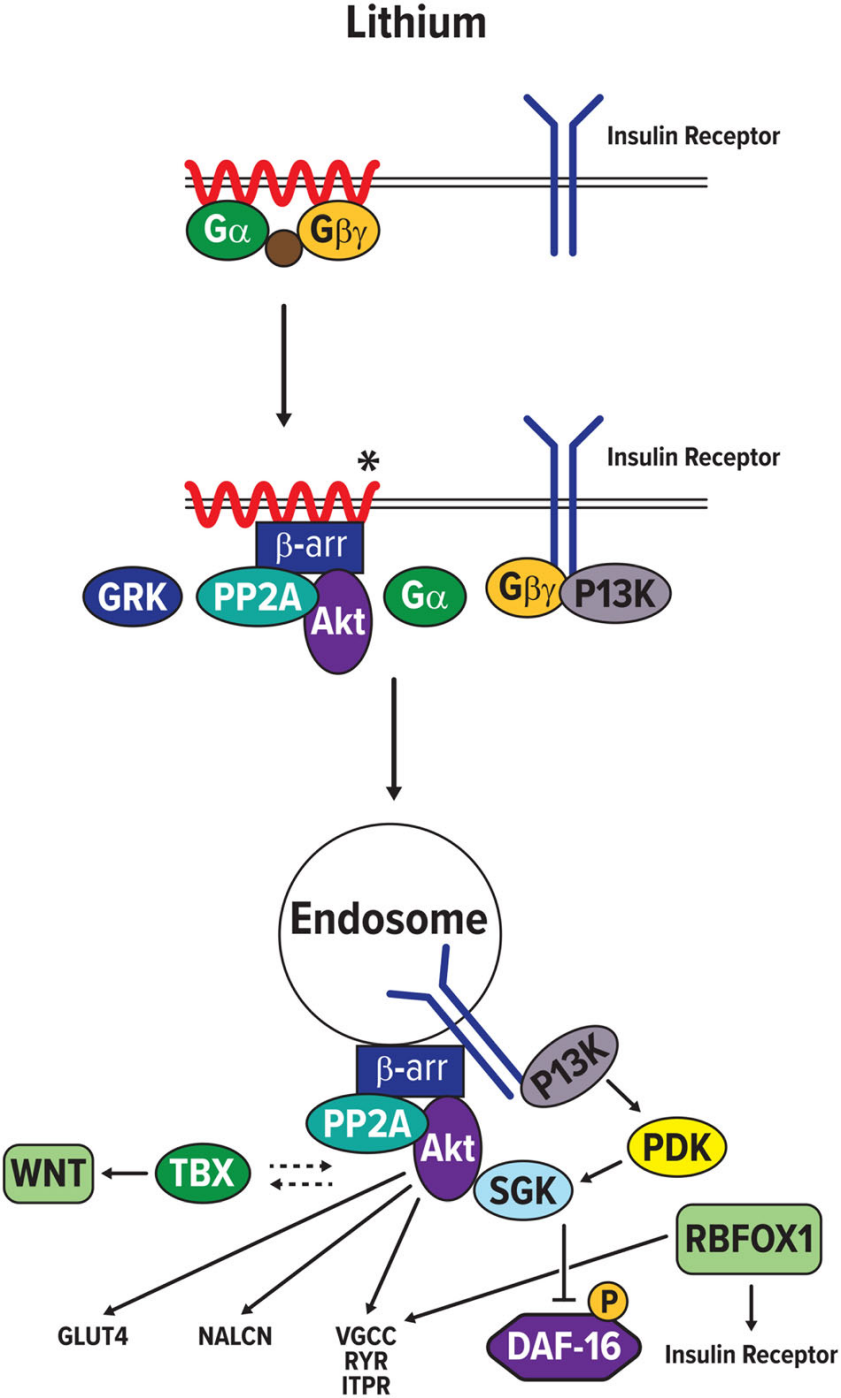
Lithium signaling pathway and related targets identified in *C. elegans.* Lithium affected DAF-16 (Forkhead box O; FOXO) transcription factor phosphorylation via a pathway that required the insulin receptor, G proteins, β-arrestin, PDK-1, Akt, and SGK-1. Akt/SGK-1 can regulate the function of various ion channels by potentially phosphorylating consensus sites in voltage-gated calcium channels (VGCC), the ryanodine receptor (RYR), the Na^+^ leak current channel (NALCN) and the inositol 1,4,5 trisphosphate receptor (ITPR2). The T-box transcription factor (TBX) interacts with Wnt and insulin signaling pathways. RNA binding protein, *fox-1* homolog (RBFOX1) regulates the splicing of VGCC and insulin receptor genes, among others.

Previously, we used molecular genetics approaches to explore non-traditional targets of antipsychotic drugs. We reported that the drugs activated Akt in both cultured neuronal cells [3] and *C. elegans* [4]; the latter finding was discovered independently by Buttner and colleagues [5]. Furthermore, we determined that clozapine stood apart from other antipsychotic drugs in terms of its molecular targets, and that lithium resembled clozapine in this regard [2]. Drugs were evaluated for their ability to cause phosphorylation of a Forkhead box O (FOXO) protein in *C. elegans*, DAF-16, thus preventing its entry into the nucleus in response to short-term starvation. Using various genetic mutant strains, we showed that the insulin receptor gene (*daf-2*) and G proteins were required for activation of Akt via phosphoinositide-dependent kinase-1, PDK-1 [2]. In stark contrast to most of the antipsychotics, clozapine and lithium also required β-arrestin and serum- and glucocorticoid-inducible kinase-1 (*sgk-1*) to affect DAF-16. The lithium pathway that we originally constructed is shown in modified form in Fig. 1 and is remarkable for the overlap with the top 25 reprioritized genes identified by Ou et al. The similarities are striking, although they require corroboration, and they furnish additional validation for using *C. elegans* in psychiatry research. This approach is further supported by observations that risk genes for schizophrenia, bipolar disorder and major depression are highly conserved during evolution, including expression in *C. elegans* [6, 7]. The various risk gene sets are enriched for genes that are essential for life [6, 7], which may help to explain their evolutionary conservation.

Ou et al. propose that lithium targets PI3K-Akt signaling to address the deficits in neuronal growth cone formation/neurite outgrowth that are associated with BD. This is a sound conclusion based on previous work and the biology of PI3K-Akt signaling [3]. The ultimate molecular targets are still unclear, but additional clues from *C. elegans* may be relevant. The insulin signaling pathway maintains pharyngeal pumping during starvation and requires the function of *pdk-1*, Akt, β-arrestin and various calcium-related channels [8] (Fig. 1). We reported that the ryanodine receptor, L-type calcium channels, and the Na^+^ leak current channel (NALCN) and its subunits contain consensus phosphorylation sites for Akt/SGK [8] as does the inositol 1,4,5-trisphosphate receptor (ITPR2). It is noteworthy that CACNA1C, NALCN, and ITPR2 have previously been identified as putative risk genes in bipolar disorder [9]. Additional research supports a relationship between calcium and bipolar disorder [10]; the observations mentioned here directly link calcium channel function to the PI3K-Akt signaling pathway.

Of course, PI3K-Akt is activated by additional signals besides insulin. Other growth factors, including NGF and BDNF, employ this pathway and transactivation of growth factor receptors via G proteins may also be important [11, 12].

More recently, lithium was found to improve goal-directed behavior in a model of diminished motivation in some *C. elegans* mutants, but not others [13]. In fact, loss-of-function mutations in *tbx-2*—the *C. elegans* counterpart of the TBX20 transcription factor, a putative suicide risk gene—caused diminished motivation that significantly worsened with lithium treatment [13]. *tbx-2* mutations also affect dauer formation, which is a special developmental stage in *C. elegans* induced by starvation and crowding akin to hibernation. The dauer response is regulated by insulin signaling and *pdk-1*. In addition, TBX transcription factors have been implicated in cancer by regulating Wnt expression [14] (see Fig. 1), which also fits with the emerging story from the work of Ou and colleagues. As expected, lithium could not improve motivation in *pdk-1* loss-of-function mutants because *pdk-1* is required for lithium-induced activation of Akt. Another mutant, *fox-1*, was unresponsive to lithium, although the reason is currently unknown. Interestingly, the human ortholog of *fox-1* is RBFOX1, a risk gene for bipolar disorder [9]. RBFOX1 also affects splicing of the insulin receptor gene [15] and a voltage-gated calcium channel (VGCC) [16] as indicated in Fig. 1. It is worth mentioning that lithium could restore goal-directed behavior in mutants with loss-of-function mutations in genes such as RGS7, DCC (*unc-40*) and two kinases—BRSK (*sad-1*) and HIPK (*hpk-1*)—possibly regulated by Akt. Therefore, *C. elegans* has potential value for exploring the pharmacogenetics of lithium and other CNS drugs.

We have previously discussed how the discovery of connections between clozapine, lithium, and SGK in *C. elegans* may have important clinical implications. These drugs are noted for inducing diabetes insipidus [17]. Lithium’s nephrotoxic actions may stem, in part, from its effects on the epithelial Na^+^ channel (ENaC) because ENaC has previously been implicated by Trepiccione and Christensen [18], and it is regulated by SGK [19]. The relationship between clozapine, lithium, and SGK1 may also be relevant for the increased incidence of hypertension in patients treated with these drugs. Lastly, clozapine may produce superior therapeutic benefits because of its hybrid nature. It acts on the same major targets as other antipsychotic drugs, while also sharing functional activity with lithium (e.g., activating SGK) that other antipsychotics lack.

Molecular genetic studies in *C. elegans*, GWAS and network analysis converge on the PI3K-Akt pathway as a significant mediator of the response to lithium treatment. The molecular studies add key mechanistic insights that complement the statistical genomics approaches and, when taken together, increase our confidence in the validity of the findings. Moreover, recent studies in *C. elegans* have provided new candidate targets of lithium that agree with existing models. Finally, the growing impact of *C. elegans* and other model organisms on psychiatry research should receive wider attention.
